# Experiences of Inclusion and Exclusion Across the Gynaecological Cancer Continuum for Individuals With Diverse Gender and Sexuality Backgrounds: A Systematic Review

**DOI:** 10.1002/pon.70518

**Published:** 2026-06-08

**Authors:** Sally‐Anne Boding, Stephanie Newton Webb, Tamara Butler, Amanda Hutchinson, Laura Hanlon, Hayley Russell

**Affiliations:** ^1^ Adelaide University Magill Australia; ^2^ Yardhura Walani National Centre for Aboriginal and Torres Strait Islander Wellbeing Research Australian National University Canberra Australia; ^3^ Grief Australia Victoria Australia

**Keywords:** cancer screening, cervical cancer, exclusion, health equity, inclusion, LGBTQIA+, oncology, socio‐ecological model, supportive care, systematic review

## Abstract

**Objective:**

Despite growing research on cancer care and survivorship among LGBTQIA+ populations, studies specific to gynaecological cancer care remain limited. This review explores experiences of inclusion and exclusion in gynaecological cancer care services for LGBTQIA+ individuals.

**Methods:**

A systematic search was conducted in August 2024, retrieving 4000 results, of which 15 met the inclusion criteria. Critical appraisal was conducted using the Mixed Methods Appraisal Tool, and a convergent integrated approach was applied with narrative synthesis of findings according to the socio‐ecological model of health.

**Results:**

All articles focussed on cervical screening. Experiences of inclusion and exclusion across intrapersonal, interpersonal, and organisational levels related to the socio‐ecological model. At the intrapersonal level, lack of individual knowledge and gender dysphoria contributed to exclusion, while access to information and supportive screening experiences fostered inclusion. Interpersonal factors associated with exclusion were inadequate provider knowledge, poor communication, lack of sensitivity and fear of speculum insertion due to experiences of pain, while inclusion was associated with strong patient‐provider relationships and knowledgeable, affirming care. At the organisational level, exclusion stemmed from binary intake forms, gendered environments, insurance issues, and absence in heath resources, while inclusive strategies, such as non‐gendered waiting rooms, inclusive materials, and appropriate insurance coverage supported engagement.

**Conclusion:**

Improved health literacy, healthcare provider knowledge, and welcoming clinical environments are key to inclusive cervical screening experiences for LGBTQIA+ individuals. Despite the initial aim to review care across the gynaecological cancer continuum, available studies focussed only on cervical screening. Research is urgently needed to inform inclusive practices across all tumour types and stages of gynaecological cancer care.

## 1 Background

Gynaecological cancers affect the reproductive and sexual organs,^1^ can substantially impact individuals' quality of life, and contribute to the global health care burden [1]. In 2022, gynaecological cancers accounted for 15.25% of all cancer cases in women worldwide and it is predicted that by 2050, new diagnoses will increase by almost 50% [1]. Despite this, gynaecological cancer research is vastly underfunded when compared to other tumour strains, leading to continual unmet needs for patients [2]. Further, gynaecological cancer statistics are not disaggregated to those who identify as lesbian, gay, bi‐sexual, transgender, queer, non‐binary or asexual (hereafter, respectfully referred to as LGBTQIA+). This gap is likely due to the lack of systematic collection of gender or sexuality status in health records or cancer registries [3].

Individuals who identify as LGBTQIA+ are often medically underserved and at higher risk of receiving sub‐standard cancer treatment and survivorship care [4]. Additionally, LGBTQIA+ populations have higher rates of tobacco and alcohol use [4], unprotected sex, and incidence of obesity [5], and lower levels of health insurance, health literacy, and screening behaviours. All these factors correlate with increased risk of gynaecological cancer diagnoses, particularly cervical and endometrial cancers [2, 4, 6, 7, 12]. Critically, this increase in cancer risk is not caused by LGBTQIA+ status, but by a heightened exposure to structural barriers that can increase cancer risk for all those who experience them, regardless of gender or sexuality [8]. For LGBTQIA+ people, chronic and prolonged exposure to discrimination and stigma, known as minority stress, has been linked to an increase in the development of chronic illnesses, such as cancer [9], while also significantly contributing to decreased psychological and physical wellbeing [8, 10, 11]. The structural barriers contributing to minority stress and impeding health equity can include institutional practices, lack of health services, media, and resources that constrain the opportunities for physical and psychological wellbeing of LGBTQIA+ people [12, 13]. These barriers are further compounded through economic instability and difficulty accessing secure income, inequitable employment, discrimination within housing, increased experiences of poverty, and a lack of health insurance all leading to inequitable service provision and uptake [9, 14, 15].

### 1.1 Experiences of Cancer Care

For LGBTQIA+ individuals, the experience of structural barriers and increased minority stress can result in a reluctance to access cancer services due to previous experiences of stigma and discrimination within cancer care [12, 16, 17], psychological difficulties through all stages of diagnosis and treatment, including post‐traumatic stress and depression [17, 18], and isolation due to programs and supportive services not being inclusive of LGBTQIA+ people or their caregivers [16]. Negative and discriminatory experiences in cancer care can also be exacerbated by a lack of provider knowledge about specific needs for LGBTQIA+ populations [6, 17]. Gynaecological cancer, while stigmatised itself within the general population [19], may be more so for LGBTQIA+ populations. This may be due to differing needs related to sexual and reproductive health and issues surrounding gender incongruence and dysphoria among transmasculine individuals [12, 20, 21], all of which can contribute to lower help seeking behaviours and contribute to later stage diagnosis [22]. Moreover, there is critical need for research that investigates the experiences of LGBTQIA+ populations within gynaecological cancer care to enable the development of supportive programs and equitable gynaecological cancer care provision [12]; by synthesising the existing evidence, this review hopes to address this need.

### 1.2 Socio‐Ecological Model

Social ecological models acknowledge that individuals are situated within the social world and systems and that these systems underpin health outcomes [23]. These models assume that this level of influence occurs not only on multiple levels, but that these levels are interactive and reinforcing [23, 24]. This way of understanding the interaction between health outcomes and the intersection of societal structures is the core of the socio‐ecological model in which an individual's behaviour impacts, and is impacted by their place within their community, and social world [24, 25]. The socio‐ecological model poses that there are five hierarchical levels that are nested within each other and include intrapersonal, interpersonal, organisational, community, and policy levels [24, 25, 26] all of which contribute to the way an individual views their own health and accesses health care services. The socio‐ecological model offers a more comprehensive and holistic understanding of the way in which exclusion and inclusion within gynaecological cancer care are experienced within LGBTQIA+ populations.

Therefore, we aimed to systematically review the existing evidence pertaining to the experiences of inclusion and exclusion for LGBTQIA+ individuals within gynaecological cancer care with the hope of increasing knowledge regarding equitable care and improving patient outcomes.

## 2 Methods

### 2.1 Sources and Search Strategy

This systematic review was conducted in accordance with the Preferred Reporting Items for Systematic Reviews and Meta‐Analysis (PRISMA) guidelines [27] and the protocol registered with PROSPERO (CRD42024510689). Guided by the PICo framework [28], we developed a comprehensive search strategy for published studies on the experiences of exclusion and inclusion of LGBTQIA+ people with gynaecological cancers (see Table 1). Six databases were included in the search: Web of Science, Embase, Medline, Scopus, PsycINFO and Nursing and Allied Health (see Supporting Information S1 for all search strategies). Searches were conducted in August 2024.

**TABLE 1:** PICo search strategy.

P Population/problem	I Interest	Co Context
(“Gender minorit*” or transgender or trans‐gender or non‐binary or “sexual minorit*” or lesbian* or Bi‐sexual or gay or queer or LGBTQA* or bisexual or pansexual or asexual) AND (Endometr* or uter* or cervi* or ovar* or vulva* or vag* or gynae* or gyne) and (cancer* or neoplas* or carcinoma* or malignan* or tumour* or Tumour*)	(Stigma or exclusion or bias or inclusion or barrier* or facilitator* or Survivo*)	(Hospital* or Facilit* or “cancer care” or hospice or rehabilitation or service* or unit* or screening or care or prevent* or provision or palliative or support or “support group”)

*Note:* Boolean operators and/or were used to narrow or expand, respectively, the search.

### 2.2 Article Eligibility Criteria

All peer reviewed, qualitative, quantitative, and mixed methods studies, published in English between January 2009 and August 2024, were included. This timeframe was chosen to increase likelihood that the outcomes of included papers would be relevant to people's current experiences. Studies were included that reported the experiences of exclusion, which the authors defined as experiences that reduce or deny service uptake; or inclusion which the authors defined as experiences that engage or increase service uptake in gynaecological cancer care. Participants needed to be aged 18 years and above who had either been screened, diagnosed, treated, or were in palliative care, and who identified with one or more of the following: gay, lesbian, bisexual, pansexual, queer, gender diverse, asexual, non‐binary or transgender men. Exclusion criteria included studies reporting outcomes in those under 18 years of age, studies published before 2009 or not in English. Studies focusing on transgender women, those not born with female reproductive organs, and cisgender men were excluded as were reviews (including meta‐analysis), opinion pieces, grey literature, conference proceedings, editorials, presentations, case studies, book chapters, and theses.

### 2.3 Screening and Data Collection

Search results were uploaded to Covidence [29] where duplicates were removed. Titles and abstracts were screened by the primary researcher (SB) with full text review completed by two authors (SB/SW) in accordance with the inclusion and exclusion criteria. No conflicts were identified. The following information was extracted from each included study: authors, year of publication, country, participant demographics, design and methodology, study objectives, healthcare setting or support service, results (qualitative and quantitative), and recommendations, as relevant to experiences and feelings of inclusion or exclusion within supportive care services. For consistency, data from two studies were first independently extracted (SB/AH) and compared. After consensus on data extraction was reached, the remaining data were extracted by one reviewer (SB).

### 2.4 Risk of Bias Assessment

Two reviewers (SB/LH) appraised each study independently using the most recent version of the mixed methods appraisal tool (MMAT) [30]. The MMAT is a critical appraisal tool designed for the assessment of qualitative, quantitative and mixed methods studies and allows for the appraisal of most common study methodologies and designs. Before methodological appraisal, the MMAT asks two screening questions, 1. Does the study have clear research questions? 2. Do the collected data address the research questions? For the study to qualify for inclusion within this review, both questions needed to be ‘yes’, which all studies appraised did. Following this, each study underwent an evaluation of five criteria with each obtaining an overall quality score where 20% is allocated per criterion met, therefore, 0% represents no criterion met, with 100% meaning all criteria met. Through this process scores ranged from 40% to 100% and consensus was reached unanimously (see Supporting Information S1 for MMAT results).

### 2.5 Synthesis

The data were analysed using a convergent integrated approach consistent with the Joanna Briggs Institute guidelines for synthesising mixed methods results [31]. Due to the heterogeneity of the interventions and outcomes, meta‐analysis was not possible. A narrative synthesis was conducted with the socio‐ecological model of health used to organise the integrated findings at three levels—intrapersonal, interpersonal, and organisational. Findings were organised into the socio‐ecological model based on the way in which exclusion and inclusion were experienced by the studies' participants. That is, the intrapersonal level incorporated the way personal knowledge and attitudes towards cervical screening excluded or included individuals in relation to screening uptake; the interpersonal level included experiences of inclusion and exclusion when experienced through interacting with others such as communication with healthcare providers throughout the screening trajectory; and the organisational level included décor of clinical spaces and insurance coverage acted as inclusive or exclusionary. Through the incorporation of this model a more comprehensive and holistic understanding of cervical cancer screening can be presented thereby showcasing the way in which experiences at various levels can influence feelings of inclusion or exclusion and service uptake.

## 3 Results

**FIGURE 1:**
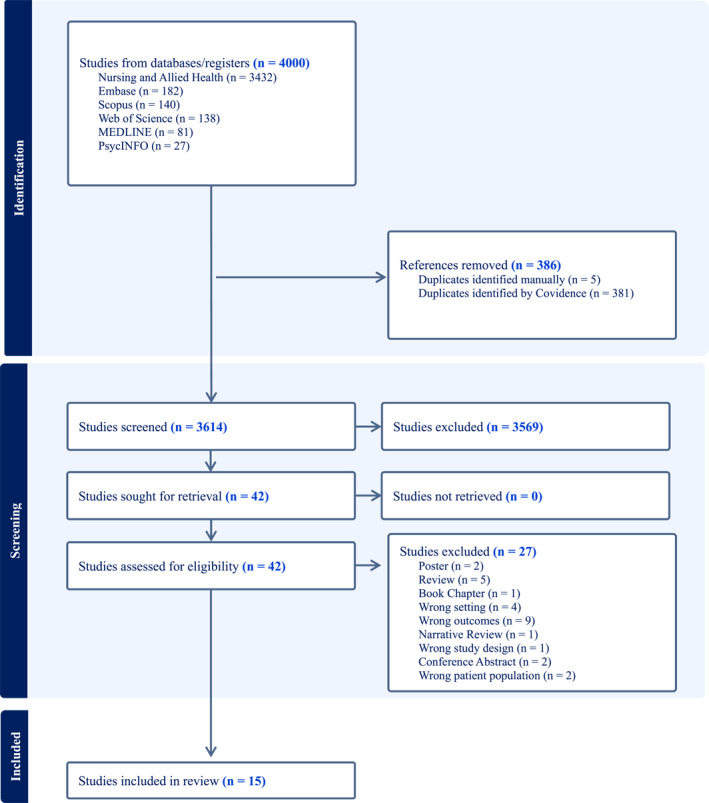
Prisma diagram.

The search identified 4000 articles. After the removal of 386 duplicates, title and abstract screening identified 42 studies eligible for full text review. Of these 27 were excluded for a variety of reasons (see Figure 1 for PRISMA diagram) but was most often because the study did not report on experiences of inclusion or exclusion. Reference lists of included articles were also checked for additional potentially relevant studies but yielded no results, leaving 15 included articles. No studies were excluded based on MMAT results.

### 3.1 Characteristics of Included Studies

**FIGURE 2:**
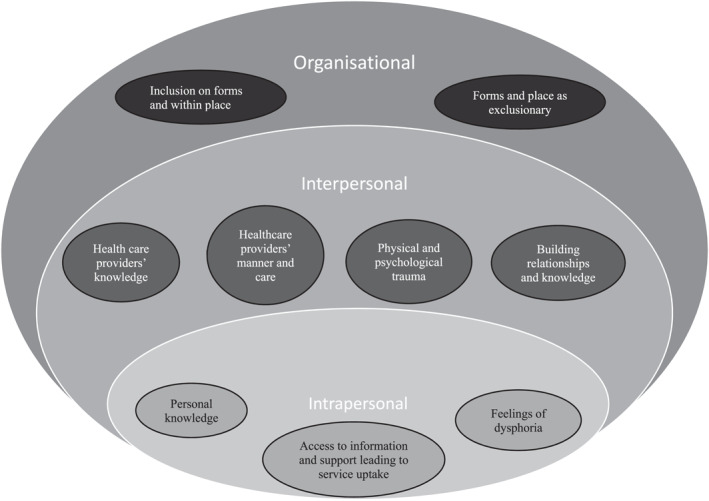
Experiences of exclusion and inclusion within gynaecological cancer services for LGBTQIA+ people structured by the socio‐ecological model.

Included studies were published between 2010 [32] and 2023 [33] with nine studies conducted in the USA [21, 34, 35, 36, 37, 38, 39, 40, 41], three in Australia [33, 42, 43], one in the UK [44], one in Aotearoa New Zealand [45] and one in Canada [32] (see Table 2 for full characteristics). All studies (*n =* 15) were related to the pap smear test. Through narrative synthesis, integrated findings were organised according to the socio‐ecological model [24] with 9 main experiences of exclusion and inclusion identified at the intrapersonal, interpersonal and organisational levels (see Figure 2).

**TABLE 2:** Characteristics of included studies.

Author/Year	*N*	Country	Sexual orientation	Gender identity	Design	Method	Cancer care continuum	Reference
Agénor/2015	18	USA	Lesbian 9; Bi‐sexual 5; queer 5, gay women 2; another sexual identity 1	Masculine 1; feminine 10, androgynous 2; gender queer 1; another gender expression 2; not sure 2	Qualitative focus groups	Inductive/deductive thematic analysis	Cervical cancer screening	[35]
Agénor/2016	32	USA	Not recorded	Male 11; transgender man, trans man, female‐to‐male, transmasculine 22; genderqueer 6;	Qualitative Semi‐structured interviews	Grounded theory	Cervical cancer screening	[36]
Johnson/2016	226	USA	Lesbian (68%); Bi‐sexual (14.6%); queer (17.5%)	Female (84.5%); FTM or genderqueer (15.5%)	Convergent‐parallel mixed methods design	Survey	Cervical cancer screening	[37]
Johnson/2016	20	USA	Lesbian/gay (11/55%); queer (6/30%); Bi‐sexual (3/15%)	Female (16/80%); trans man (4/20%)	Qualitative—semi structured interviews and open ended‐questions	Content analysis (inductive/deductive)	Cervical cancer screening	[38]
Johnson/2020	20	USA	Queer 12 (60%), gay/Lesbian 6 (30%), bisexual 2 (10%)	Transgender 9 (45%); genderqueer 8 (40%); gender non‐conforming 3 (15%)	Qualitative—Semi structured interviews	Content analysis (inductive/deductive socioecological model lens)	Cervical cancer screening	[39]
Paschen‐Wolff/2020	22	USA	Lesbian/gay 17 (77.3%); bisexual 4 (18.2%); pansexual 1 (4.5%)	Women 22	Qualitative—semi structured interviews	Content analysis/descriptive approach	Cervical cancer screening	[40]
Peitzmeier/2017	32	USA	Not recorded	Male 11 (34%); transgender man, trans man, female‐to‐male, transmasculine 22 (69%); genderqueer 6 (19%)	Qualitative—semi‐structured interviews	Modified grounded theory (code book)	Cervical cancer screening	[22]
Peitzmeier/2020	32	USA	Not recorded	Male 11 (34%); transgender man, trans man, female‐to‐male, transmasculine 22 (69%); genderqueer 6 (19%)	Qualitative—semi‐structured interview	Modified grounded theory, codebook comprised of inductively derived codes	Cervical cancer screening	[41]
Polek/2010	96	USA	Lesbian, bisexual	Not recorded	Survey	Descriptive correlational	Cervical cancer screening	[42]
Curmi/2016	9	Australia	Lesbian	Women	Qualitative Semi structured interviews	Qualitative descriptive design	Cervical cancer screening	[44]
Kerr/2022	196	Australia	Not reported	Trans men 76 (38.8%); gender diverse 120 (61.2%); genderqueer 20 (10.2%); genderfluid 10 (5.1%).	Quantitative Survey	Descriptive and multiple regression analyses	Cervical cancer screening	[43]
Kerr/2023	2424	Australia	Lesbian or gay 1047 (43.3%); bisexual 510 (21.1%); pansexual 194 (8%); queer 464 (19.2%); asexual 82 (3.4%); something else 122 (5%)	Cisgender woman 1866 (77.4%); trans man 144 (6%); non‐binary 402 (16.7%)	Survey	Multivariate regression analysis	Cervical cancer screening	[34]
Berner/2021	137	UK	Not recorded	Transmasculine 109; non‐binary 24; other non‐cisgender identities 3	Cross Sectional/Mixed methods	Quantitative—descriptives, Fisher's Exact test; qualitative—reflexive thematic analysis	Cervical cancer screening	[45]
Caroll/2023	318	Aotearoa	Not recorded	Trans man 129; non‐binary (assigned female at birth) 189	Mixed methods	χ2 tests and content analysis	Cervical cancer screening	[46]
McIntyre/2010	7	Canada	Lesbian 7	Women 7	Qualitative Semi‐structured interview	Conventional qualitative analysis	Cervical cancer screening	[33]

### 3.2 Experiences of Exclusion and Inclusion at the Intrapersonal Level

#### 3.2.1 Personal Knowledge

Eleven studies examined individual knowledge regarding what pap/cervical screening is testing for, why this might be important, and how this acted as a barrier to service uptake [32, 33, 35, 36, 37, 39, 41, 42, 43, 44, 45]. For transgender men not attending screening due to their gender [37, 44] was noted, whereas both transgender and sexuality diverse women reported not attending due to not engaging in penetrative sexual intercourse, contributing to a perception that they were at low risk of HPV transmission [32, 35, 39, 41, 42, 43, 45].

#### 3.2.2 Feelings of Dysphoria

Seven studies reported feelings of gender dysphoria experienced by transgender men and how this impacted on ability or willingness to undergo screening tests, leading to experiences of severe psychological distress [15, 21, 36, 38, 40, 44, 45]. This distress was associated not only with physical screening and vulnerability in displaying of bodies [40, 44], as well as incongruence with identifying as male while having to undergo a female or feminised testing [21].

#### 3.2.3 Access to Information and Support Leading to Service Uptake

Eleven studies reported that individual knowledge regarding screening, positive past experiences and feelings of support influenced service uptake [21, 32, 33, 35, 36, 37, 39, 40, 41, 42, 45]. For some transgender men service uptake was increased due to a perceived increased risk of cervical cancer from testosterone usage [35], re‐gendering their bodies in terms of language [21] and viewing the pap test as a personal responsibility [40]. Family history of cancer [21, 35, 36] and previous abnormal results were also facilitators of screening [37] for both sexuality and gender diverse populations. Other factors included living in inner suburbs [42], being older [32, 42], having access to information (such as online resources) as well as friends and family to discuss health matters with and support people to attend tests with [32, 35, 36].

### 3.3 Experiences of Exclusion and Inclusion at the Interpersonal Level

#### 3.3.1 Exclusion due to Pain and Fear

Prior experiences of pain or fear of pain related to penetration of the speculum, regardless of LGBTQIA+ expression, acted as a barrier to engaging in cervical screening [21, 37, 43, 44, 45] or led to assumptions that the test would be emotionally traumatic [42]. Two studies reported distress and discomfort for lesbian, transgender and non‐binary individuals when discussing elements of the screening with health care providers (HCPs) [32, 45]. Other reasons LGBTQIA+ individuals avoided screening services included previous experiences of distrust and discrimination within and beyond the health care system [36, 38] as well as a lack of trust in, or access to a regular provider [21, 43, 45].

#### 3.3.2 Physical and Psychological Trauma

Eight studies found experiences of abuse were a barrier to service uptake [21, 32, 36, 38, 40, 43, 44, 45]. Abuse or trauma was experienced in participants' personal lives [38], a health service, or a combination of both [36, 44, 45]. Any experience of trauma led to a reduction in regular screening participation. These experiences of post‐traumatic stress were further compounded by pressure from HCPs to undergo screening tests [40] regardless of feeling safe and/or providing consent, resulting in both psychologically and physically traumatic experiences [21, 32].

#### 3.3.3 Health Care Providers' Knowledge

Eleven articles explored healthcare providers' knowledge about the cervical screening needs of LGBTQIA+ people and how these led to experiences of exclusion or acted as a barrier to screening uptake. This occurred through dissuading individuals from undergoing cervical screening because of their sexuality [32, 44], or not asking nor prompting individuals to undergo cervical screening tests [34, 35, 39, 42, 43, 44]. A lack of knowledge by HCPs regarding the risks and needs of LGBTQIA+ participants [32, 34, 36, 39, 40, 41, 44] was also noted. Other experiences of exclusion included feminising cervical screening by HCPs using anatomical names for genitalia (vagina, e.g.) as well as using feminised terms for clothing [40]. Individuals also discussed needing to educate their HCPs on transgender issues [21].

Relatedly, HCPs assumptions of heterosexuality led to individuals being forced to disclose sexuality or gender, as identified in eight studies [32, 33, 34, 36, 38, 41, 42, 43], to ensure they were receiving correct and relevant information. These experiences prevented open communication with HCPs and created frustration about having to explain their gender or sexuality and practices [38, 43] and included both inappropriate and unprofessional behaviour.

Further, both sexuality and gender diverse individuals who reported good relationships with their provider still discussed HCPs assumptions regarding sexuality [32, 34] leading to a lack of disclosure [37, 41, 42].

#### 3.3.4 Healthcare Provider's Manner and Care

HCPs manner and care was found to impact on the experiences of individuals in nine studies [21, 32, 34, 37, 40, 42, 43, 44, 45]. HCPs reportedly made individuals feel uncomfortable, on display or violated when discussing screening tests and their bodies [40, 44] and sexual practices [43].

Related to manner and care, HCPs dismissing experiences of pain for transgender men was found in three studies [21, 40, 44], along with notable power differentials between patient and provider [40]. This was apparent in HCPs assuming individuals' gender identity and sexuality through the use of incorrect pronouns and making heteronormative assumptions, leading to reduced feelings of autonomy over decision making [37, 40]. Changes in HCP/patient relationship dynamics following disclosure of sexuality were also noted [43], along with patients feeling both physically and psychologically unsafe when engaging within screening services along with the anticipated or experienced incidences of discrimination [15, 34, 44, 45] for both sexuality and gender diverse populations.

#### 3.3.5 Building Relationships and Knowledge to Provide Quality Care

An association between HCP knowledge and open and trusting relationships was identified in 12 studies [21, 33, 34, 35, 36, 37, 38, 39, 40, 42, 43, 44]. Specifically, HCPs not assuming sexuality or gender, showing patience, compassion, and providing non‐judgmental advice was important to individuals regardless of sexuality or gender identity [36, 38, 43, 44]. The correct use of pronouns and including partners and support persons [21, 38], having a regular general practitioner for both sexuality diverse [33, 37, 43] and gender diverse populations, [21, 33, 38, 44], knowledge of specific needs [34, 35, 36, 37, 39, 42], and seeing HCPs that were the same gender, sexuality, and ethnicity [34] all contributed to experiences of inclusion and screening uptake. The importance of HCPs educating themselves on the screening experiences, needs, and wants of LGBTQIA+ individuals, and deconstructing their own prejudices and discriminatory practices was emphasised [21, 33, 34, 36, 37, 38, 39, 40, 42, 43, 44, 45], as was being offered the opportunity to self‐swab [35, 44]. This increased knowledge facilitated caring, respectful, and trusting relationships and was linked to increased service uptake and improved psychological wellbeing in 10 studies [21, 33, 34, 36, 37, 38, 39, 40, 42, 43, 44, 45].

### 3.4 Experiences of Exclusion and Inclusion at the Organisational Level

#### 3.4.1 Exclusion Through Forms and Place

Experiences within healthcare were related to research, advertising, admission forms, and physical places (such as waiting rooms) for all gender and sexuality populations, as noted in 11 studies [21, 34, 35, 36, 37, 38, 39, 40, 42, 43, 44, 45]. Eight studies reported experiences of exclusion through cisgendered, heteronormative advertising and health promotion, overly feminine waiting rooms within health care services [21, 34, 36, 37, 39, 40, 43, 44], and binary intake forms that do not include options for sexuality diversity [34, 36, 39]. Further, for Black lesbian, queer, and bisexual individuals, exclusion was also experienced through healthcare providers not reflecting their identities [34].

Barriers to cervical screening for transgender men and gender‐diverse individuals have been documented across multiple studies, highlighting a range of systemic and structural challenges. Four American studies noted that insurance companies would not cover the costs of screening tests of transgender men if their gender marker changed to male, with some individuals having already experienced out‐of‐pocket expenses [21, 36, 38, 45]. However, other studies noted how individuals required pap testing to receive gender affirming surgery such as hysterectomy or access to testosterone [21, 42]. Further, not sending reminders for a pap test to transgender men due to their change in gender identity or being misgendered through recall letters [44, 45] was also of note. Two studies noted admission and intake forms did not include variants beyond a male/female binary [40, 44], with other studies reporting experiences of exclusion through lack of research, particularly inclusive of transgender men [35, 44], and fear of the impact of United States government policies on the transgender community [38].

Finally, it was noted that those who identified as LGBTQIA+ and were from a non‐English speaking background, lived in remote or regional towns, had a disability (regardless of severity), or had not attended a screening service in the last 12 months, all reported lower odds of participating in cervical screening [33]. These studies underscore how intersecting factors such as insurance exclusions, restrictive intake processes, cisnormative environments, research gaps, and broader sociopolitical influences contribute to the inequities faced by LGBTQIA+ populations in accessing cervical screening.

#### 3.4.2 Inclusion Within Forms and Place

Inclusion within screening services and increased service uptake was reflected through LGBTQIA+ orientations and identities being included on intake forms, in the decor of the practice including displaying of anti‐discrimination posters and policies [21, 34, 36, 37, 38, 39, 43, 44, 45], and the availability of specific LGBTQIA+ health services and practices [21, 33, 34, 36, 38, 40, 43, 44].

## 4 Discussion

This review was conducted to synthesise published evidence on the experiences of exclusion and inclusion throughout the cancer care continuum, from screening to post‐treatment and palliative care within gynaecological cancer care. However, the search strategy yielded only studies pertaining to cervical cancer screening therefore, this review offers valuable insights into how LGBTQIA+ individuals experience exclusion and inclusion in cervical screening services. These insights are integral to understanding the barriers and facilitators to cervical screening uptake, which has been reported as lower within LGBTQIA+ populations [5]. Integrated findings were organised according to the socio‐ecological model [24] with 9 main experiences of exclusion and inclusion identified at the intrapersonal, interpersonal, and organisational levels. The reviewed studies demonstrate the way in which inclusion or exclusion within screening services are impacted by LGBTQIA+ knowledge surrounding cervical screening and HCP knowledge, open and clear communication between HCP and patient, and feelings of safety and psychological wellbeing offered through and by HCP and services across individual, interpersonal and organisational levels. A table of recommendations has been provided to enable clear action points to increase cervical screening uptake at multiple levels of the socio‐ecological model for LGBTQIA+ populations (see Table 3).

An important outcome of this review is the level of dysphoria, pain, and fear experienced by transgender men due to the cervical screening process [21, 36, 37, 40, 44]. The severity of these outcomes was strongly influenced by HCP attitude, level of knowledge, and care. This demonstrates the critical need for HCPs to be not only knowledgeable regarding transgender patients' fears and previous experience within cervical screening, but also to facilitate sensitive, safe, and knowledgeable care. Lesbian and non‐binary individuals also reported high levels of distress, pain, and reduced screening uptake due to fears about the insertion of the speculum, particularly if they had not engaged in penetrative sex or if the procedure was insufficiently explained by a HCP, which is consistent with other underserved and minoritised population experiences [46]. This highlights the need for HCPs to be knowledgeable regarding experiences of pain and discomfort for all individuals seeking cervical screening, particularly as some people may choose not to disclose their LGBTQIA+ identity or their sexual history. Adopting this approach enables service provision that is both inclusive and individualised, occurring collaboratively and respectfully through offering pain management, information regarding the procedure, and options for differing specula sizes to maximise initial and repeat service uptake.

Routinely offering self‐swabbing as an alternative to traditional methods of HCP collection should also be provided to all individuals regardless of gender or sexuality, as the findings from this review only reported on transgender men [35, 44]. Research suggests that self‐swabbing is a safe, effective, and easy method and is the preferred option for many populations where trauma or religion are barriers to collection [46, 47]. This method has recently been endorsed by the US Food and Drug administration [48], and self‐swabbing programs have been created in places such as Canada and Australia [42, 49, 50] which provide comprehensive information and resources for underserved populations and LGBTQIA+ people specifically. Moreover, self‐swabbing has shown to increase feelings of empowerment, is more cost effective, has the ability to reach underserved populations and those with intersecting identities [51]. To increase self‐swabbing uptake within the LGBTQIA+ community, implementing LGBTQIA+ navigators and engagement with local LGBTQIA+ health and community organisations can help promote self‐swabbing through information and resource sharing from trusted organisations and peers [51]. Insights may also be drawn from colorectal cancer screening research where the impact of emotional barriers, particularly fear and disgust associated with the faecal occult blood test, are informing efforts to improve screening uptake [52].

Offering this alternative and equally effective screening method may also foster increased feelings of collaboration, healthcare self‐determination, and ultimately increase screening uptake within LGBTQIA+ populations. This can also help foster a strong sense of self‐determination and advocacy, which is imperative to increasing service uptake in the face of HCPs denying, dissuading, or providing misinformation regarding the need for cervical screening, which was evident in the results of this review [32, 34, 36, 39, 40, 41, 44]. Empowering individuals to advocate for their healthcare through a strong knowledge base, self‐confidence [32] and through the empowerment of knowledge and community support can help reduce the spread of misinformation in service provision.

Furthermore, access to correct information is vital to health literacy. In turn, this can reduce fear and pain and increase feelings of autonomy, decision making power, and service uptake [53]. This applies equally for knowledge about what HPV is and how it is transmitted, which was found to be severely lacking within the populations in the included studies. Improving health literacy is important to increase screening uptake and thus lowering the incidence of cervical cancer diagnosis [41, 53, 54]. To improve health literacy, pamphlets [21, 37] and public health campaigns should be created for LGBTQIA+ people (e.g., “Own It” campaign in Australia [50]) worldwide. The use of promotional material to increase cervical screening uptake has been shown to be successful [54], therefore, promotion of cervical screening guidelines at a national level should be implemented though various mediums (e.g., social media, advertising, television, and radio).

A critical finding of this review is the influence HCPs have over the experiences, outcomes, and future screening behaviours of LGBTQIA+ individuals. This was most apparent in relation to HCP knowledge, care, and acceptance. Strong HCP knowledge about diversity of needs and being open and collaborative in care improved psychological wellbeing and facilitated screening uptake [21, 34, 43, 55]. This included not assuming gender and/or sexuality through asking open‐ended questions and having knowledge about population‐specific risk factors and increased needs related to cervical screening. Other factors of inclusion were having access to a regular HCP, and living in an urban location [21, 33, 38, 43, 44]. This is consistent with other studies related to LGBTQIA+ populations, experiences of disclosure, and HCP acceptance [16, 56]. An affirming HCP response to identity disclosure resulted in higher satisfaction and increased compliancy to HCP recommendations, a finding which is consistent with Ussher et al. [57]. Individuals were less likely to disclose their identity and more likely to report poor outcomes when HCPs assumed sexuality and/or gender, or made offensive and unprofessional comments regarding their bodies and/or sexual practices [21, 34, 43]. Other deterrents included experiences of, or anticipation of exclusion, stigma, violence or discrimination, as previous research has also noted [36, 39, 42]. This demonstrates that HCPs ignorance, bias, and/or a lack of knowledge about LGBTQIA+ individuals leads not only to an increase in abuses of power, denial of care, and withholding of gender affirming interventions [40], but can also increase incidences of psychological distress and experiences of exclusion within screening services. These findings emphasise the responsibility of HCPs to be educated on the needs of LGBTQIA+ populations through organisational training to ensure they can provide not only information, but also affirming care, resulting in experiences of inclusion, safety, and acceptance within the cervical screening setting.

One concept to help facilitate these relationships is through the practice of cultural humility. Cultural humility involves committing to life‐long learning through self‐reflection and self‐critique, with an emphasis on where one is situated culturally, assessing assumptions, bias, attitudes, and areas for growth [58, 59]. This can help reduce power imbalances and create open and empathetic communication [58, 59, 60]. Cultural humility also involves authentic engagement with beliefs, attitudes, and values that are different from one's own which is important as LGBTQIA+ individuals often intersect with other minoritised populations such as cultural, ethnic, low income, or rural populations [15, 59]. Furthermore, having clinics specifically designed for, and to meet the needs of LGBTQIA+ people could help safeguard against deeply ingrained and resistant prejudice that can be embedded within political and ideological belief systems held by some HCPs. This review has noted an increase in service uptake when LGBTQIA+ specific clinics were available [21, 33, 34, 36, 38, 40, 43, 44]. Designated clinics and spaces can enable LGBTQIA+ people to feel more confident that they will be receiving knowledgeable, competent, inclusive, and humility informed personalised care in a safe and welcoming environment, thereby increasing service uptake.

Finally, healthcare clinics are uniquely positioned to provide welcoming and safe environments for LGBTQIA+ individuals. Congruently, with previous research in other tumour streams [55], the display of anti‐discrimination policies, rainbow flags, and stickers supporting LGBTQIA+ people provide visual markers for inclusion and acceptance for these populations [36, 37, 45, 57]. However, the review found that waiting and treatment rooms at times reflected overly femininised décor which meant transgender men felt unwelcome and experienced increased feelings of dysphoria. Because cervical screening has been traditionally considered a woman's exam [21] this review has highlighted the way in which the décor and clinic itself can enhance feelings of inclusion through neutral décor, gender neutral bathrooms, and the placement of brochures and information sheets that are specific to LGBTQIA+ individuals [21, 34, 40, 43, 44]. This, along with options for LGBTQIA+ identity to be recorded on intake forms [21, 32, 33, 34, 39, 42, 45], are vital in encouraging individuals to participate in screening recommendations in spaces that make them feel safe and included.

An integral finding of this review relates to the lack of research beyond cervical screening for LGBTQIA+ people, highlighting a critical need across the gynaecological cancer care continuum. The dearth of studies indicates that not only does more research need to be conducted within this area, but that the conducting of large‐scale research should be made a priority as majority of studies included within this review were qualitative with smaller sample sizes. While qualitative research is imperative to guiding changes in healthcare, larger scale quantitative data can demonstrate the depth and breadth of this need, along with differences and needs across population groups, thereby directing policy needs and changes to enable and direct funding [3, 5, 33]. To further facilitate this, LGBTQIA+ identity needs to be captured within cancer registries to be able to inform policy through the delineating of which LGBTQIA+ populations may be at higher risk for specific cancers, thereby informing what services are needed to support individuals, their carers, and families through the cancer trajectory and beyond [3, 61].

It is important to acknowledge the valuable research that has been published since the initial searches of this review were conducted in 2024. Since this time three studies met the inclusion criteria, with two studies relating to experiences of inclusion and exclusion within cervical screening specifically for transgender individuals [62], those from low‐income settings [63], and one relating to those with an experience of uterine cancer [22]. These studies strengthen and corroborate the findings of the current review with exclusion being driven by poor HCP education, cisgendered and heteronormative assumptive resources and HCP discourse, fear of penetration, and fear of disclosure [22, 62, 63]. A lack of insurance and a regular healthcare provider [63] added further barriers, as did gendered spaces being exclusionary [22], the cost of testing, lack of transportation, as well as issues related to poor mental and physical health and forgetting, or not thinking about cervical screening [63].

### 4.1 Clinical Implications/Recommendations

Based on the review's findings and those of the individual studies, the following recommendations have been made utilising the socio‐ecological model and incorporating desired outcomes of these recommendations on LGBTQIA+ experiences of inclusion within cervical screening services (see Table 3). At the interpersonal level recommendations to improve cervical screening experiences include discussing self‐swabbing options [21, 33, 42, 44, 45], which should be routinely offered to help reduce experiences of fear, discrimination, and stigma, and increase bodily autonomy and collaborative health care partnerships [33, 40, 42, 44]. This, coupled with active listening, taking time to describe the procedure and offering self‐speculum insertion for each client [40] would build rapport, trust, and increase screening uptake. At the organisational level, information should be tailored to LGBTQIA+ populations to increase health literacy [21, 32, 33, 34, 39, 42, 45] with ongoing education for HCPs, along with practicing cultural humility to enable a reduction in prejudice, cisgendered assumptions, and substandard care [57]. Ensuring waiting rooms and spaces are LGBTQIA+ welcoming through the inclusion of signage, ensuring staff are culturally competent, intake forms are inclusive of gender and sexuality [21, 32, 33, 34, 39, 42, 45], and appropriate insurance authorisation prior to screening appointments [21, 40, 42] will endeavour to create inclusive cervical screening services for all LGBTQIA+ populations at the organisational level. Finally, at the community level, inclusion within public health campaigns specifically targeted to LGBTQIA+ populations, and accessible information regarding the needs of LGBTQIA+ populations for cervical screening, and promotion of screening guidelines [21, 33, 39, 45, 50] is imperative, along with the inclusion of sexuality and gender diversity to be captured within cancer registries [3, 61] and implementation of healthcare clinics specifically for LGBTQIA+ people [21, 33, 34, 36, 38, 40, 43, 44].

**TABLE 3:** Recommendations at the socio‐ecological level and desired outcomes.

Socio‐ecological level	Outcomes
Interpersonal level– Knowledge and empowerment about speculum insertion and self‐swabbing	To reduce experiences of fear and pain, active listening to individual concerns and experiences should occur in the first instance [22, 42]. HCPs should be routinely offering self‐swabbing as an alternate to HCP collection [22, 34, 43, 45, 46]. Explain the steps before commencing screening, allowing individuals to insert speculum themselves, and empowering individual to stop at any time [41]. Ask individuals their preferred terms for anatomy to reduce dysphoria (canal for vagina, for example) [41].
Organisational level– Improved healthcare provider knowledge leading to improved care	Continual HCP education to improve knowledge base regarding cervical screening for LGBTQIA+ individuals. Gain knowledge regarding experiences of discrimination and abuse some individuals have encountered both outside and within healthcare settings. This can help with HCPs prejudice and cisgendered/normative assumptions and provide empathetic care. Strong knowledge of LGBTQIA+ risk factors for cervical cancer and needed exams allows for the sharing of information which can improve health literacy of LGBTQIA+ people. HCPs should engage with cultural humility and commit to life‐long learning through self‐reflection, self‐critique, and dispelling assumptions, bias, negative attitudes to provide safe and inclusive care [58].
	It would be beneficial for HCPs to be trained in trauma informed care due to higher incidence of sexual assault within LGBTQIA+ populations.
Organisational level– Clinic environments should create feelings of safety for LGBTQIA+ individuals	LGBTQIA+ welcoming signage, pamphlets, posters, and anti‐discrimination policies should be displayed in all healthcare facilities [22, 33, 34, 35, 40, 43, 46]. Train frontline staff in cultural competency, as well as training into the use of pronouns and preferred names [22]. Make gender neutral bathrooms available [22]. Improve inclusivity of intake forms, avoiding cisgender assumptions and include questions about sexuality [22, 33, 34, 35, 36, 37, 38, 39, 40, 41, 43, 44, 45, 46]. For clinics and countries that utilise private or insurance covered healthcare systems, ensure that appropriate authorisation is received to avoid denial of coverage for cervical screening services [22, 41, 43].
	Provide specific information tailored for LGBTQIA+ populations (such as transgender men or lesbian populations for example), as well as information regarding the needs and experiences specific to the wider population [22, 33, 34, 35, 40, 43, 46].
Community level– Improving health literacy for LGBTQA+ individuals	Inclusion of LGBTQIA+ navigators and engagement with local LGBTQIA+ health and community organisations to promote self‐swabbing through information and resource sharing from trusted organisations [51]. Public health campaigns should be implemented to increase awareness of LGBTQIA+ screening needs [22, 34]. Promotion of cervical screening guidelines at a national level should be implemented though various mediums (such as advertising, television, and radio for example) as in Australia [50]. Specific information regarding screening options, who should be screened, when, and how often can increase screening uptake, as well as normalise and destigmatise people from LGBTQIA+ populations obtaining screening tests [40, 46].
	Inclusion of sexuality and gender diversity to be captured within cancer registries [3, 62]
Creating feelings of safety through designation of specialised clinics	Increasing the number of LGBTQIA+ specific cancer clinics to reduce experiences of stigma and discrimination and improve service uptake [22, 34, 35, 37, 39, 41, 44, 45].

## 5 Limitations

The limitations of this review are twofold. Firstly, although it has provided useful and important details of the lived experiences of LGBTQIA+ individuals within cervical screening services, the aim was to synthesise experiences of exclusion and inclusion within gynaecological cancer support and care services throughout the gynaecological cancer care continuum. This was not possible, however highlights an immense gap in the research as studies detailing these outcomes were scarce (at the time of screening). Research is needed to understand how individuals connect with, utilise, and experience gynaecological cancer care beyond screening and to ensure inclusion, appropriateness of service delivery, and that information is being sufficiently and appropriately communicated to LGBTQIA+ individuals throughout the cancer trajectory. A final limitation is related to the search strategy and the possible inclusion of more comprehensive terms to capture the breadth of screening and prevention programs and interventions, particularly related to self‐swabbing and HPV vaccination. As this is a growing area of knowledge, future research should investigate the experiences of inclusion and exclusion within these modalities.

## 6 Conclusion

This review highlights the significant influence that HCP and clinical environments can have on the inclusion or exclusion of LGBTQIA+ individuals within cervical cancer screening services. Although the original aim was to synthesise research from across the gynaecological cancer care continuum, available research focussed exclusively on cervical cancer screening. Integrated findings structed using the socio‐ecological model reveal that inclusive care is experienced when HCP have adequate knowledge, communicate clearly, and listen to individual's concerns, as well as provide welcoming clinical environments. However, exclusion is felt when assumptions of sexuality or gender are made, experiences of discrimination occur, incorrect information regarding testing needs is provided, or when individuals concerns and needs are dismissed. This can lead to increased experiences of dysphoria and stigma, reduced screening uptake and poorer health outcomes. Addressing these barriers through HCP education, cultural humility, and inclusive public health messaging is critical to improve health literacy and outcomes for LGBTQIA+ populations. Critically, this review identified an urgent need for research that examines experiences of inclusion and exclusion across the gynaecological cancer continuum (beyond cervical screening) for LGBTQIA+ individuals to ensure consistent provision of safe, relevant, and inclusive care.

## Funding

S.‐A.B. and L.H. were supported by a cost‐of‐living stipend to complete research as part of a PhD thesis, in the form of an Australian Government Research Training Programme Scholarship. T.B. was supported by National Health and Medical Research Council (NHMRC) Investigator Grant (#2008097). S.N.W., A.H. and H.R. did not receive any specific grant from funding agencies in the public, commercial, or not‐for‐profit sectors.

## Conflicts of Interest

The authors declare no conflicts of interest.

## Supporting information


Supporting Information S1

## Data Availability

Data sharing not applicable to this article as no datasets were generated or analysed during the current study.

## Endnotes

^1^
These cancers include vulvar, vaginal, cervical, uterine, ovarian, and fallopian tube cancer.
