# Supplementary material for: Experiences of Inclusion and Exclusion Across the Gynaecological Cancer Continuum for Individuals With Diverse Gender and Sexuality Backgrounds: A Systematic Review
Source: Psychooncology. 2026 Jun 8;35(6):e70518. doi: 10.1002/pon.70518 (PMC13247537; doi:10.1002/pon.70518)
Supplement: Supplementary file 1 — Supporting Information S1 [file PON-35-e70518-s001.docx]

**Supplementary Table S1**

*Full Search Strategy and Databases*

*Web of Science*

("Gender Minorit*" or Transgender or Trans-gender or Non-Binary or “Sexual Minorit*” or Lesbian* or Bi-sexual or Gay or Queer or LGBTQA* or Bisexual or Pansexual or Asexual) AND (Endometr* or Uter* or Cervi* or Ovar* or Vulva* or Vag* or Gynae* or Gyne) and (Cancer* or Neoplas* or Carcinoma* or Malignan* or Tumour* or Tumor*) AND (Stigma or Exclusion or Bias or Inclusion or Barrier* or Facilitator* or Survivo*) AND (Hospital* or Facilit* or “Cancer Care” or Hospice or Rehabilitation or Service* or Unit* or Screening or Care or Prevent* or Provision or Palliative or Support or “Support Group”)

*Embase*

("Gender Minorit*" or Transgender or Trans-gender or Non-Binary or “Sexual Minorit*” or Lesbian* or Bi-sexual or Gay or Queer or LGBTQA* or Bisexual or Pansexual or Asexual) AND (Endometr* or Uter* or Cervi* or Ovar* or Vulva* or Vag* or Gynae* or Gyne) and (Cancer* or Neoplas* or Carcinoma* or Malignan* or Tumour* or Tumor*) AND (Stigma or Exclusion or Bias or Inclusion or Barrier* or Facilitator* or Survivo*) AND (Hospital* or Facilit* or “Cancer Care” or Hospice or Rehabilitation or Service* or Unit* or Screening or Care or Prevent* or Provision or Palliative or Support or “Support Group”)

*Medline*

("Gender Minorit*" or Transgender or Trans-gender or Non-Binary or “Sexual Minorit*” or Lesbian* or Bi-sexual or Gay or Queer or LGBTQA* or Bisexual or Pansexual or Asexual) AND (Endometr* or Uter* or Cervi* or Ovar* or Vulva* or Vag* or Gynae* or Gyne) and (Cancer* or Neoplas* or Carcinoma* or Malignan* or Tumour* or Tumor*) AND (Stigma or Exclusion or Bias or Inclusion or Barrier* or Facilitator* or Survivo*) AND (Hospital* or Facilit* or “Cancer Care” or Hospice or Rehabilitation or Service* or Unit* or Screening or Care or Prevent* or Provision or Palliative or Support or “Support Group”)

*Scopus*

("Gender Minorit*" or Transgender or Trans-gender or Non-Binary or “Sexual Minorit*” or Lesbian* or Bi-sexual or Gay or Queer or LGBTQA* or Bisexual or Pansexual or Asexual) AND (Endometr* or Uter* or Cervi* or Ovar* or Vulva* or Vag* or Gynae* or Gyne) and (Cancer* or Neoplas* or Carcinoma* or Malignan* or Tumour* or Tumor*) AND (Stigma or Exclusion or Bias or Inclusion or Barrier* or Facilitator* or Survivo*) AND (Hospital* or Facilit* or “Cancer Care” or Hospice or Rehabilitation or Service* or Unit* or Screening or Care or Prevent* or Provision or Palliative or Support or “Support Group”)

*PsycINFO*

("Gender Minorit*" or Transgender or Trans-gender or Non-Binary or “Sexual Minorit*” or Lesbian* or Bi-sexual or Gay or Queer or LGBTQA* or Bisexual or Pansexual or Asexual) AND (Endometr* or Uter* or Cervi* or Ovar* or Vulva* or Vag* or Gynae* or Gyne) and (Cancer* or Neoplas* or Carcinoma* or Malignan* or Tumour* or Tumor*) AND (Stigma or Exclusion or Bias or Inclusion or Barrier* or Facilitator* or Survivo*) AND (Hospital* or Facilit* or “Cancer Care” or Hospice or Rehabilitation or Service* or Unit* or Screening or Care or Prevent* or Provision or Palliative or Support or “Support Group”)

*Nursing and Allied Health*

("Gender Minorit*" or Transgender or Trans-gender or Non-Binary or “Sexual Minorit*” or Lesbian* or Bi-sexual or Gay or Queer or LGBTQA* or Bisexual or Pansexual or Asexual) AND (Endometr* or Uter* or Cervi* or Ovar* or Vulva* or Vag* or Gynae* or Gyne) and (Cancer* or Neoplas* or Carcinoma* or Malignan* or Tumour* or Tumor*) AND (Stigma or Exclusion or Bias or Inclusion or Barrier* or Facilitator* or Survivo*) AND (Hospital* or Facilit* or “Cancer Care” or Hospice or Rehabilitation or Service* or Unit* or Screening or Care or Prevent* or Provision or Palliative or Support or “Support Group”)

**Supplementary Table S2**

*Mixed Methods Appraisal Tool (MMAT) Quality Appraisal Outcomes*

*All articles passed the initial screening questions S1 and S2, this table summarises assessment items only.*

| **Author** | **Agenor et al., 2015** | Responses | | | |
| --- | --- | --- | --- | --- | --- |
|  |  | Yes | No | Can’t tell | Comments |
| **1. Qualitative** | - 1. Is the qualitative approach appropriate to answers the research question? | X |  |  |  |
|  | - 1. Are the qualitative data collection methods adequate to address the research question? | X |  |  |  |
|  | - 1. Are the findings adequately derived from the data? | X |  |  |  |
|  | - 1. Is the interpretation of results sufficiently substantiated by data? | X |  |  |  |
|  | - 1. Is there coherence between qualitative data sources, collection, analysis and interpretation? | X |  |  |  |
| **Author** | **Agenor et al., 2016** | | | | |
| **1. Qualitative** | - 1. Is the qualitative approach appropriate to answers the research question? | X |  |  |  |
|  | - 1. Are the qualitative data collection methods adequate to address the research question? | X |  |  |  |
|  | - 1. Are the findings adequately derived from the data? | X |  |  |  |
|  | - 1. Is the interpretation of results sufficiently substantiated by data? | X |  |  |  |
|  | - 1. Is there coherence between qualitative data sources, collection, analysis and interpretation? | X |  |  |  |
| **Author** | **Johnston et al., 2016** |  | | | |
| **1. Qualitative** | - 1. Is the qualitative approach appropriate to answers the research question? | X |  |  |  |
|  | - 1. Are the qualitative data collection methods adequate to address the research question? | X |  |  |  |
|  | - 1. Are the findings adequately derived from the data? | X |  |  |  |
|  | - 1. Is the interpretation of results sufficiently substantiated by data? | X |  |  |  |
|  | - 1. Is there coherence between qualitative data sources, collection, analysis and interpretation? | X |  |  |  |
| 1. **Quantitative descriptive** | - 1. Is the sampling strategy relevant to address the research question? | X |  |  |  |
|  | - 1. Is the sample representative of the target population? |  |  | X |  |
|  | - 1. Are the measurements appropriate? | X |  |  |  |
|  | - 1. Is the risk of nonresponse bias low? | X |  |  |  |
|  | - 1. Is the statistical analysis appropriate to answer the research question? | X |  |  |  |
| 1. **Mixed methods** | - 1. Is there an adequate rationale for using a mixed methods design to address the research question? | X |  |  |  |
|  | - 1. Are the different components of the study effectively integrated to answer the research question? | X |  |  |  |
|  | - 1. Are the outputs of the integration of qualitative and quantitative components adequately interpreted? | X |  |  |  |
|  | - 1. Are divergences and inconsistencies between quantitative and qualitative results adequately addressed? | X |  |  |  |
|  | - 1. Do the different components of the study adhere to the quality criteria of each tradition of the methods involved? | X |  |  |  |
| **Author** | **Johnston et al., 2016** |  | | | |
| **1. Qualitative** | - 1. Is the qualitative approach appropriate to answers the research question? | X |  |  |  |
|  | - 1. Are the qualitative data collection methods adequate to address the research question? | X |  |  |  |
|  | - 1. Are the findings adequately derived from the data? | X |  |  |  |
|  | - 1. Is the interpretation of results sufficiently substantiated by data? |  | X |  |  |
|  | - 1. Is there coherence between qualitative data sources, collection, analysis and interpretation? | X |  |  |  |
| **Author** | **Johnston et al., 2020** |  | | | |
| **1. Qualitative** | - 1. Is the qualitative approach appropriate to answers the research question? | X |  |  |  |
|  | - 1. Are the qualitative data collection methods adequate to address the research question? | X |  |  |  |
|  | - 1. Are the findings adequately derived from the data? | X |  |  |  |
|  | - 1. Is the interpretation of results sufficiently substantiated by data? | X |  |  |  |
|  | - 1. Is there coherence between qualitative data sources, collection, analysis and interpretation? | X |  |  |  |
| **Author** | **Paschen-wolff et al., 2020** |  | | | |
| **1. Qualitative** | - 1. Is the qualitative approach appropriate to answers the research question? | X |  |  |  |
|  | - 1. Are the qualitative data collection methods adequate to address the research question? | X |  |  |  |
|  | - 1. Are the findings adequately derived from the data? | X |  |  |  |
|  | - 1. Is the interpretation of results sufficiently substantiated by data? | X |  |  |  |
|  | - 1. Is there coherence between qualitative data sources, collection, analysis and interpretation? | X |  |  |  |
| **Author** | **Peitzmeir et al., 2017** |  | | | |
| **1. Qualitative** | - 1. Is the qualitative approach appropriate to answers the research question? | X |  |  |  |
|  | - 1. Are the qualitative data collection methods adequate to address the research question? | X |  |  |  |
|  | - 1. Are the findings adequately derived from the data? | X |  |  |  |
|  | - 1. Is the interpretation of results sufficiently substantiated by data? | X |  |  |  |
|  | - 1. Is there coherence between qualitative data sources, collection, analysis and interpretation? | X |  |  |  |
| **Author** | **Peitzmeier et al., 2019** |  | | | |
| **1. Qualitative** | - 1. Is the qualitative approach appropriate to answers the research question? | X |  |  |  |
|  | - 1. Are the qualitative data collection methods adequate to address the research question? | X |  |  |  |
|  | - 1. Are the findings adequately derived from the data? | X |  |  |  |
|  | - 1. Is the interpretation of results sufficiently substantiated by data? | X |  |  |  |
|  | - 1. Is there coherence between qualitative data sources, collection, analysis and interpretation? | X |  |  |  |
| **Author** | **Polek & Hardie, 2010** |  | | | |
| 1. **Quantitative descriptive** | - 1. Is the sampling strategy relevant to address the research question? | X |  |  |  |
|  | - 1. Is the sample representative of the target population? |  |  | X |  |
|  | - 1. Are the measurements appropriate? | X |  |  |  |
|  | - 1. Is the risk of nonresponse bias low? |  |  | X |  |
|  | - 1. Is the statistical analysis appropriate to answer the research question? | X |  |  |  |
| **Author** | **Curmi et al., 2015** |  | | | |
| **1. Qualitative** | - 1. Is the qualitative approach appropriate to answers the research question? | X |  |  |  |
|  | - 1. Are the qualitative data collection methods adequate to address the research question? | X |  |  |  |
|  | - 1. Are the findings adequately derived from the data? | X |  |  |  |
|  | - 1. Is the interpretation of results sufficiently substantiated by data? | X |  |  |  |
|  | - 1. Is there coherence between qualitative data sources, collection, analysis and interpretation? | X |  |  |  |
| **Author** | **Kerr et al., 2022** |  | | | |
| 1. **Quantitative descriptive** | - 1. Is the sampling strategy relevant to address the research question? | X |  |  |  |
|  | - 1. Is the sample representative of the target population? |  | X |  |  |
|  | - 1. Are the measurements appropriate? | X |  |  |  |
|  | - 1. Is the risk of nonresponse bias low? |  |  | X |  |
|  | - 1. Is the statistical analysis appropriate to answer the research question? | X |  |  |  |
| **Author** | **Kerr et al., 2023** |  | | | |
| 1. **Quantitative descriptive** | - 1. Is the sampling strategy relevant to address the research question? | X |  |  |  |
|  | - 1. Is the sample representative of the target population? |  |  | X |  |
|  | - 1. Are the measurements appropriate? | X |  |  |  |
|  | - 1. Is the risk of nonresponse bias low? |  |  | X |  |
|  | - 1. Is the statistical analysis appropriate to answer the research question? | X |  |  |  |
| **Author** | **Berner et al., 2021** |  | | | |
| **1. Qualitative** | - 1. Is the qualitative approach appropriate to answers the research question? | X |  |  |  |
|  | - 1. Are the qualitative data collection methods adequate to address the research question? | X |  |  |  |
|  | - 1. Are the findings adequately derived from the data? | X |  |  |  |
|  | - 1. Is the interpretation of results sufficiently substantiated by data? | X |  |  |  |
|  | - 1. Is there coherence between qualitative data sources, collection, analysis and interpretation? | X |  |  |  |
| 1. **Quantitative descriptive** | - 1. Is the sampling strategy relevant to address the research question? | X |  |  |  |
|  | - 1. Is the sample representative of the target population? |  | X |  |  |
|  | - 1. Are the measurements appropriate? | X |  |  |  |
|  | - 1. Is the risk of nonresponse bias low? |  |  | X |  |
|  | - 1. Is the statistical analysis appropriate to answer the research question? | X |  |  |  |
| 1. **Mixed methods** | - 1. Is there an adequate rationale for using a mixed methods design to address the research question? | X |  |  |  |
|  | - 1. Are the different components of the study effectively integrated to answer the research question? | X |  |  |  |
|  | - 1. Are the outputs of the integration of qualitative and quantitative components adequately interpreted? | X |  |  |  |
|  | - 1. Are divergences and inconsistencies between quantitative and qualitative results adequately addressed? | X |  |  |  |
|  | - 1. Do the different components of the study adhere to the quality criteria of each tradition of the methods involved? | X |  |  |  |
| **Author** | **Carroll et al., 2023** |  | | | |
| **1. Qualitative** | - 1. Is the qualitative approach appropriate to answers the research question? |  |  | X |  |
|  | - 1. Are the qualitative data collection methods adequate to address the research question? |  |  | X |  |
|  | - 1. Are the findings adequately derived from the data? | X |  |  |  |
|  | - 1. Is the interpretation of results sufficiently substantiated by data? | X |  |  |  |
|  | - 1. Is there coherence between qualitative data sources, collection, analysis and interpretation? | X |  |  |  |
| 1. **Quantitative descriptive** | - 1. Is the sampling strategy relevant to address the research question? |  |  | X |  |
|  | - 1. Is the sample representative of the target population? |  |  | X |  |
|  | - 1. Are the measurements appropriate? | X |  |  |  |
|  | - 1. Is the risk of nonresponse bias low? | X |  |  |  |
|  | - 1. Is the statistical analysis appropriate to answer the research question? |  |  | X |  |
| 1. **Mixed methods** | - 1. Is there an adequate rationale for using a mixed methods design to address the research question? | X |  |  |  |
|  | - 1. Are the different components of the study effectively integrated to answer the research question? | X |  |  |  |
|  | - 1. Are the outputs of the integration of qualitative and quantitative components adequately interpreted? | X |  |  |  |
|  | - 1. Are divergences and inconsistencies between quantitative and qualitative results adequately addressed? | X |  |  |  |
|  | - 1. Do the different components of the study adhere to the quality criteria of each tradition of the methods involved? | X |  |  |  |
| **Author** | **McIntyre et al., 2010** |  | | | |
| 1. **Qualitative** | - 1. Is the qualitative approach appropriate to answers the research question? | X |  |  |  |
|  | - 1. Are the qualitative data collection methods adequate to address the research question? | X |  |  |  |
|  | - 1. Are the findings adequately derived from the data? | X |  |  |  |
|  | - 1. Is the interpretation of results sufficiently substantiated by data? | X |  |  |  |
|  | - 1. Is there coherence between qualitative data sources, collection, analysis and interpretation? | X |  |  |  |
